# The potential clinical implications of extrachromosomal circular DNA as a biomarker

**DOI:** 10.1016/j.gendis.2025.101734

**Published:** 2025-06-24

**Authors:** Xuanmei Luo, Jian Cui, Hexin Li, Gang Zhao, Lihui Zou

**Affiliations:** a Clinical Biobank, Beijing Hospital, National Center of Gerontology, National Health Commission, Institute of Geriatric Medicine, Chinese Academy of Medical Sciences, Beijing 100730, China; b Peking University Fifth School of Clinical Medicine, Beijing Hospital, National Center of Gerontology, Beijing 100730, China; c Department of General Surgery, Beijing Hospital, Beijing 100730, China; d National Center of Gerontology, Institute of Geriatric Medicine, Chinese Academy of Medical Sciences, Beijing 100730, China; e The Key Laboratory of Geriatrics, Beijing Institute of Geriatrics, Institute of Geriatric Medicine, Chinese Academy of Medical Sciences, Beijing Hospital/National Center of Gerontology of National Health Commission, Beijing 100730, China

**Keywords:** Biomarker, Clinical implication, Diagnosis, Extrachromosomal circular DNA, High-throughput detection

## Abstract

Extrachromosomal circular DNA (eccDNA) is a class of circular DNA molecules that originate from and are independent of conventional chromosomes. The high specificity and stability of the circular structure of eccDNA provide new clues for its research as a disease biomarker. The recent rapid development of long-read sequencing and eccDNA identification algorithms has greatly expanded our understanding of eccDNA properties. In this review, we introduce the molecular characteristics, biological origin, and advances in detection technology of eccDNA, and mainly summarize the clinical implications of eccDNA in cancer diagnosis and prognosis, drug resistance monitoring, prenatal testing, and immune-related disease, providing support for further exploring new clinical applications of eccDNA.

## Background

Extrachromosomal circular DNA (eccDNA) is a collective term for closed circular DNA molecules that originate from but are independent of chromosomes.^1^, ^2^, ^3^, ^4^, ^5^ eccDNA was first discovered in 1964 as a series of DNA circles in mammalian cells and higher plants.^6^ Later, eccDNA has been identified in many eukaryotic species, including yeast,^7^ drosophila melanogaster,^8^ mice,^9^ hamsters,^10^ Arabidopsis,^11^ humans,^12^, ^13^, ^14^ and rice.^15^ An increasing number of studies indicate that eccDNA plays a crucial role in multiple diseases and pathological processes, particularly in cancer development and progression, immune response, and aging. In this review, we describe the state-of-the-art high-throughput technologies for eccDNA identification and discuss the potential clinical utility of eccDNA biomarkers in several diseases.

## The biogenesis and properties of eccDNA

The generation of eccDNA depends on multiple mechanisms: homologous recombination, non-homologous end joining, DNA replication, and the formation of R-loops.^1^^,^^16^ It is generally believed that eccDNA biogenesis models include the breakage-fusion-bridge cycle, chromothripsis, episome model, and translocation-deletion-amplification model.^1^^,^^17^ The size range of eccDNA is very wide, from a few hundred basepairs to several megabasepairs. Accordingly, based on size and sequence, eccDNAs can be categorized into several types (Table 1).^17^ Small polydispersed DNA enhances genomic instability.^18^ MicroDNA serves as a template for small regulatory RNA, regulating the expression level of target genes.^19^ The telomeric circle is involved in the alternative lengthening and rapid deletion of telomeres.^20^ The extrachromosomal rDNA circle contributes to aging in yeast.^21^ Notably, extrachromosomal DNA can function as mobile super-enhancers to strongly drive full-length or truncated oncogene transcription in chromosomal and extrachromosomal DNA, promoting tumor genetic heterogeneity, tumor evolution, and drug resistance.^14^^,^^22^^,^^23^ These full-length transcripts could be productively translated to increase functional protein levels. However, if these truncated transcripts contain intact binding sites for factors involved in post-transcriptional processing and translation, they can compete with normal transcripts from chromosomes for these critical factors without producing functional proteins, ultimately leading to a decrease in the expression level of corresponding proteins in the body.

**Table 1:** The classification of eccDNA.

Type	Size	Function
Small polydispersed DNA	0.1–10 kb	Involved in genomic instability
MicroDNA	100 to 400 bp	Express functional small regulatory RNAs
Telomeric circle	Integral multiples of 738 bp	Involved in the alternative lengthening and rapid deletion of telomeres
Extrachromosomal rDNA circle	19.3–40.4 kb	Contribute to aging
Extrachromosomal DNA	Several Mb	Promote tumor heterogeneity, tumor evolution, and drug resistance

## High-throughput methods for eccDNA detection

**Figure 1:**
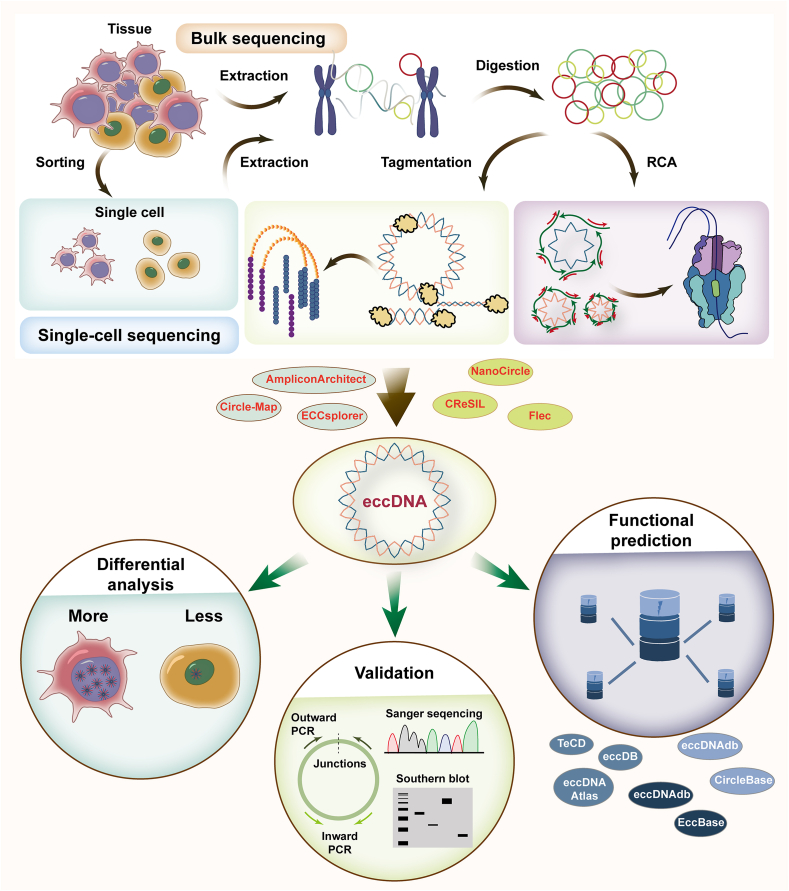
Methods in the exploration of extrachromosomal circular DNA (eccDNA). Single-cell and bulk sequencing technologies and corresponding bioinformatics algorithms have been developed for eccDNA detection. Later, validation, differential analysis, and functional prediction of eccDNA have been applied to discover disease-specific eccDNAs. RCA, rolling circle amplification; PCR, polymerase chain reaction.

Previous methods, including electron microscopy,^6^ atomic force microscopy,^24^ and fluorescence microscopy,^25^, ^26^, ^27^ were unable to provide information about eccDNA sequences. Thus, high-throughput sequencing technologies, along with the enrichment pretreatment and identification algorithms of eccDNA, have been developed (Fig. 1).

## eccDNA extraction, amplification, and sequencing

Currently, the most used method for eccDNA sequencing is Circle-Seq.^28^ The technical cornerstone of Circle-Seq lies in the fact that the circular structure of eccDNA retains high stability under Plasmid-Safe DNase digestion. Briefly, eccDNA is extracted through alkaline treatment, enriched by Plasmid-Safe DNase digestion of linear chromosomes and rolling-circle amplification (RCA), and sequenced on high-throughput sequencing platforms. To improve the efficiency of eccDNA purification, Wang et al developed a new three-step eccDNA purification (3SEP) procedure.^29^ An alkaline buffer (pH 11.8) was used to lyse cells to reduce eccDNA breakage, PacI restriction endonuclease was used to linearize mitochondrial DNA, and Solution A from either a Bingene (220501–50) or Biofargo (220501) covalently closed circular DNA purification kit selectively recovered circular DNA on magnetic silica beads.

The utilization of RCA, as well as eccDNA-safe enzyme digestion, efficiently eliminates genomic interference and ensures high-precision identification of eccDNA. However, RCA generally favors small-size eccDNA and thus introduces biases in eccDNA quantification.^30^ RCA products do not carry epigenetic information from eccDNA, resulting in the loss of crucial biological information. To overcome these limitations, Mouakkad-Montoya et al developed a strategy to enrich eccDNA without RCA.^31^ Sin et al also leveraged tagmentation reactions using the 5-mC-Tn5 transposomes and enzyme conversion to obtain epigenetic information of eccDNA molecules.^32^

eccDNA lacks centromeres and segregates randomly or asymmetrically during cell division, resulting in a high degree of eccDNA heterogeneity in daughter cells. Unfortunately, bulk sequencing methods only reveal the average features of all cells, rather than the characteristics of individual cells. Diseased cells may contain large amounts of specific eccDNA, which is highly significant for studying the selective advantages and drug resistance of eccDNA. The SMOOTH-seq and scEC&T-seq methods provide effective workflows for eccDNA detection at the single-cell level, helping analyze the cellular heterogeneity and evolution of eccDNA.^33^^,^^34^ The scEC&T-seq method enables parallel full-length mRNA sequencing. The SMOOTH-seq method enables parallel genomic variant sequencing but cannot distinguish genomic duplication variations from eccDNA events.

## eccDNA identification

AmpliconArchitect, Circle-Map, and ECCsplorer identify eccDNA from short reads,^35^, ^36^, ^37^, ^38^ but are limited by their ability to recognize genomic repetitive regions and eccDNA composed of several genomic fragments. This problem has been solved through long-read sequencing technology, which can reconstruct full-length eccDNA from RCA products more effectively than short reads without the need for sequence assembly and eccDNA prediction based on discordant or split reads.

NanoCircle, CReSIL, and Flec are developed for eccDNA identification from long reads.^12^^,^^39^^,^^40^ The Flec software only identifies eccDNA from reads containing concatemeric tandem copies, and its ability to identify large-size eccDNA is inadequate. The CReSIL tool requires high sequence coverage. The NanoCircle pipeline is based on split reads and does not require high sequence coverage, thus identifying more eccDNA events than does the CReSIL tool.

Additionally, the HolistIC tool, which integrates whole-genome sequencing and Hi-C sequencing data, is beneficial for identifying large-size eccDNA and reveals the associations between amplicons and eccDNA.^41^

## eccDNA validation and functional prediction

Conventional microscopic imaging techniques, such as electron microscopy,^6^ atomic force microscopy,^24^ and fluorescence microscopy,^25^, ^26^, ^27^ can directly visualize the circular structure of eccDNA. Electron microscopy uses electron beams for high-resolution imaging but requires a high vacuum and complex sample preparation. Two main types are scanning electron microscopy (resolution ∼1 nm) for surface morphology imaging, and transmission electron microscopy (resolution ∼0.1 nm) for thin-section ultrastructure analysis.^42^ Atomic force microscopy characterizes surface topography by directly measuring forces between the probe and the surface, allowing eccDNA imaging under physiologically relevant liquid conditions. However, this technique is constrained by slow scanning speeds and potential probe-induced eccDNA damage.^43^ Fluorescence microscopy detects fluorescent signals emitted by target-specific fluorophores upon excitation at characteristic wavelengths. This technique enables direct, real-time monitoring of eccDNA dynamics in living cells, but its spatial resolution is inherently limited by optical diffraction.^44^

The specificity of junction positions enables specific molecular detection of eccDNA biomarkers. Outward PCR, inward PCR, and Sanger sequencing can be adopted to verify the circular structure and sequence of the predicted eccDNA,^11^ while southern blotting analysis can be used as an alternative. Quantitative PCR based on outward primers is performed to verify eccDNA content.^45^

The DifCir method was developed for the differential analysis of eccDNA.^46^ Several resources provide information on the characteristics and functions of eccDNA.^47^, ^48^, ^49^, ^50^, ^51^, ^52^ For example, the eccDNA Atlas, eccDB, and TeCD provide comprehensive eccDNA repositories from multiple species; EccBase and eccDNAdb focus on eccDNA in cancer; and CircleBase and eccDNAdb are used to screen functional eccDNA and interpret molecular mechanisms.

## Applications of eccDNA as biomarkers

**Figure 2:**
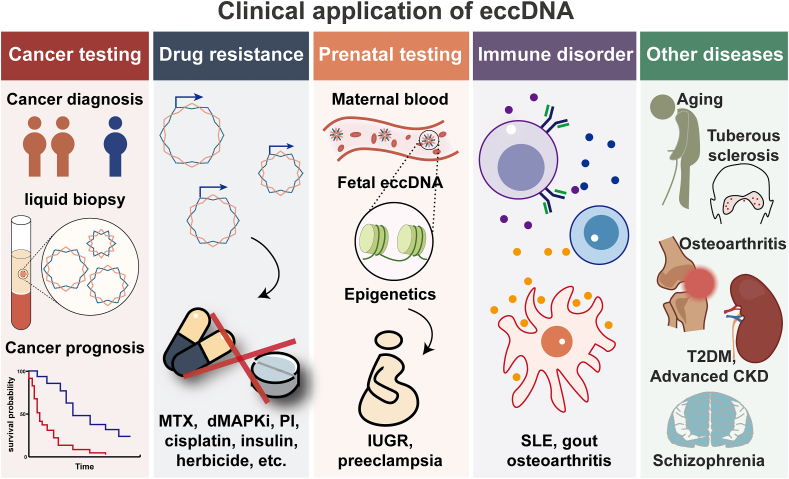
The clinical application of extrachromosomal circular DNA (eccDNA) biomarkers. eccDNA may act as biomarkers for cancer testing, monitoring drug resistance, prenatal testing, and the detection of immune disorders and other diseases. MTX, methotrexate; dMAPKi, dual MAPK inhibitors; PI, proteasome inhibitors; IUGR, intrauterine growth restriction; SLE, systemic lupus erythematosus; CKD, chronic kidney disease; T2DM, type 2 diabetes mellitus.

Since the discovery of eccDNA in mammalian cells in 1964,^6^ extensive research has been carried out on the signature spectrum of eccDNA in a variety of diseases and pathological processes,^53^^,^^54^ and its potential as a biomarker has been proposed (Fig. 2).

**Figure 3:**
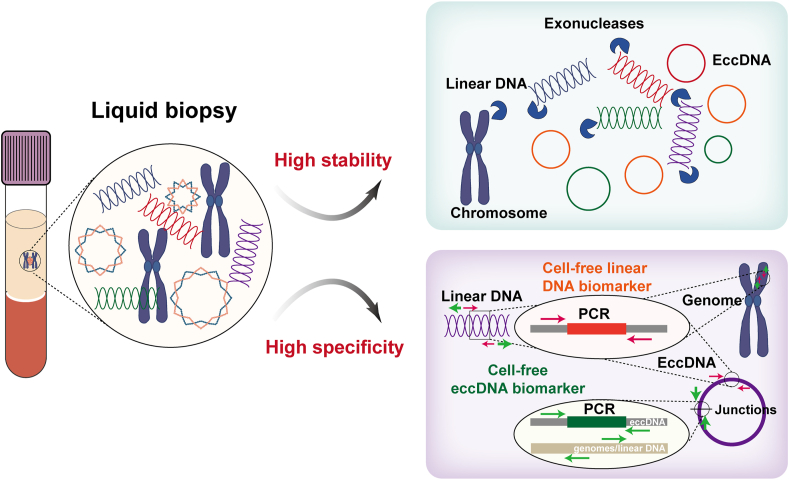
Cell-free eccDNA biomarkers have higher stability and greater specificity than cell-free linear DNA biomarkers. PCR, polymerase chain reaction.

eccDNA biomarkers exhibit exceptional stability and specificity. In terms of stability, as a closed circular structure, eccDNA is more resistant to nucleases than its linear counterparts^55^ and RNA,^56^^,^^57^ making it a more stable biomarker (Fig. 3). Notably, in liquid biopsy applications, its unique junction position and closed circular structure confer eccDNA critical specificity over cell-free linear DNA (Fig. 3). Cell-free linear DNA exists in ultra-low abundance in bodily fluids, and cell rupture (such as hemolysis) due to improper handling or storage can release large amounts of mitochondrial and chromosomal DNA from normal cells, resulting in the dilution of cell-free linear DNA signal and reduced detection sensitivity and specificity.^58^ In contrast, eccDNA biomarkers are inherently resistant to such interference: i) circular structure enables *MssI* endonuclease^13^ and exonuclease digestion to selectively eliminate mitochondrial and chromosomal DNA contamination while preserving eccDNA integrity, and ii) disease biomarkers targeting the junction position of eccDNA are inherently immune to interference from linear DNA fragments.

## eccDNA as a cancer biomarker

eccDNA serves as a potential biomarker in cancer diagnosis and management. Lung tumor tissues contain longer eccDNA than their paired normal tissues.^59^ Telomeric circles can quantify the activity of alternative lengthening of telomeres (ALT) and diagnose ALT-positive tumors.^60^^,^^61^ Luo et al systematically profiled eccDNA characteristics in multiple cancers and found that eccDNA in cancer tissues had a larger number and size than that in their paired non-cancer tissues.^62^ Additionally, eccDNA carrying specific genes had great multi-cancer diagnostic value in tissue samples, particularly when combining eccDNA carrying the *ALK* gene with eccDNA carrying the *ETV6* gene. This study defined eccDNA biomarkers based on their inclusion of exon or intron sequences, rather than traditional junction positions. Consequently, the detection of these eccDNA biomarkers necessitates high-throughput sequencing-based approaches. Furthermore, achieving sufficient data saturation is essential to both minimize confounding signals from apoptotic cell-derived eccDNA and maintain optimal detection sensitivity. Notably, a multi-cohort study revealed the presence of eccDNA in Barrett's esophagus-associated early-stage esophageal adenocarcinoma in biopsy tissues and a significant accumulation of eccDNA during the cancerous transformation of Barrett's esophagus, emphasizing that eccDNA can develop early in the transition from high-grade dysplasia to cancer and underscoring the potential of eccDNA as a pre-cancerous biomarker.^53^

Intracellular eccDNA can be released into body fluids.^59^^,^^63^^,^^64^ Therefore, cell-free eccDNA has potential applications for liquid biopsy and disease surveillance. Four types of eccDNA (eccDNA carrying the *DOCK1*, *PPIC*, *TBC1D16*, or *RP11-370A5.1* gene) were uniquely expressed in the plasma of patients with lung adenocarcinoma, which can assist in the early diagnosis of lung adenocarcinoma.^65^ However, this finding should be interpreted with caution, as the analysis relies on healthy data from publicly available databases and lacks rigorous controls. Kumar et al found that the size of eccDNA in the circulation of cancer patients decreased sharply after surgery, suggesting the application prospects of eccDNA in the dynamic monitoring of minimal residual disease.^59^

eccDNA is also of great value in assessing cancer prognosis. Cen et al found that the level of DNMT1^circle10302690-10302961^ decreased in both primary and metastatic tumors and was associated with poorer prognosis in patients with high-grade serous ovarian cancer.^66^ Other studies demonstrated that eccDNA carrying the *MYCN* gene was a responsible biomarker for adverse patient outcomes.^54^ Furthermore, Zhou et al suggested that eccDNA could lead to tumor recurrence due to its high invasiveness, radiation resistance, and mis-segregation.^67^

## eccDNA as a biomarker for drug resistance

Therapy resistance is the leading cause of death in most cancer patients. The presence of eccDNA carrying the *EGFR* gene is an indicative biomarker of resistance to radiotherapy and targeted therapy in patients with glioblastoma.^67^^,^^68^ An increase in eccDNA carrying the *DHFR* gene is associated with methotrexate-resistant cancers.^69^, ^70^, ^71^, ^72^ In melanoma, eccDNA carrying the *BRAF* gene increases resistance to dual MAPK inhibitors.^73^ Lin et al revealed that eccDNA carrying the *RAB3B* gene could promote cisplatin resistance in hypopharyngeal squamous cell carcinoma by inducing autophagy.^74^ Proteasome inhibitors are one of the most important drugs for the treatment of multiple myeloma. Wang et al revealed that eccDNA induced proteasome inhibitor resistance by amplifying the *KIF3C* gene to reduce the expression of the *MUC20* gene in multiple myeloma.^75^ Furthermore, elevated serum SORBS1^circle^ level is associated with insulin resistance in patients with type 2 diabetes mellitus.^45^ The presence of eccDNA carrying herbicide resistance genes endows plants with herbicide resistance.^76^, ^77^, ^78^

Therefore, reducing the generation of eccDNA and eliminating eccDNA that carries drug-resistant genes may be promising therapeutic strategies.^79^^,^^80^ It has been reported that dimethyl sulfoxide and hydroxyurea can reduce the amount of eccDNA in cells.^81^^,^^82^ However, these methods for eliminating eccDNA lack sequence specificity. Given that not all eccDNAs are harmful, strategies designed to target junction positions for selective elimination of specific eccDNA may be a key part of future targeted therapies. This strategy capitalizes on the highly specific positions of eccDNA biomarkers, so there is no damage to chromosomal and mitochondrial DNA, reflecting the high specificity of eccDNAs as therapeutic target markers.

## eccDNA as a biomarker for prenatal testing

eccDNA provides a new perspective for screening pregnancy-associated disorders. The eccDNA of fetal origin is widely present in the plasma of pregnant women and largely preserves the DNA methylation status of the fetal genome.^32^^,^^83^ Therefore, several serious pregnancy-associated disorders, such as preeclampsia and intrauterine growth restriction, which are associated with epigenetic alterations in the genome,^84^, ^85^, ^86^ hold promise for early screening based on relatively safe maternal blood tests. Indeed, Lin et al identified several eccDNAs in plasma that could serve as valuable non-invasive biomarkers for patients with intrauterine growth restriction.^87^ In addition, Yang et al reported the presence of eccDNA in the placenta and found that the amount of placental eccDNA in patients with intrauterine growth restriction was significantly higher than that in normal subjects.^88^ Future exploration may focus on some special eccDNA biomarkers in maternal plasma for non-invasive prenatal testing and monitoring of the therapeutic efficacy of interventions for pregnancy-associated disorders.

## eccDNAs as biomarkers for immune-related disease

eccDNA is a potent innate immunostimulant that can enhance innate immunity.^40^ Deoxyribonuclease 1-like 3 (DNASE1L3) deficiency leads to autoimmune disorders. In the plasma of patients with DNASE1L3 loss-of-function mutation, Sin et al plotted the size distribution of eccDNA and found a reduction in the first peak cluster (150–250 bp) and an increase in the second peak cluster (300–450 bp). They further calculated the value for the area under the curve (AUC) of the second peak cluster divided by the AUC value of the first peak cluster, and called it the “AUC ratio”, and proposed that it could serve as a biomarker for early diagnosis or monitoring of diseases related to DNASE1L3 deficiency.^89^ Systemic lupus erythematosus is a highly prevalent autoimmune disease associated with autoantibodies. Differential analysis of eccDNA in patients with systemic lupus erythematosus identified a set of cell-free eccDNA biomarkers associated with DNASE1L3 deficiency, with the eccDNA carrying the transcription factor *BARX2* being the most prominent.^90^

Gout is the most common inflammatory arthritis in males. Pang et al detected gout-specific eccDNAs and found that genes carried by these eccDNAs were involved in hyperuricemia, gout, and inflammation response.^91^ Osteoarthritis is a common degenerative disease of the musculoskeletal system. Xiang et al profiled eccDNA features in articular cartilage and preliminarily pointed out the potential role of eccDNA in osteoarthritis pathogenesis.^92^

## eccDNA as biomarkers for other diseases

eccDNA also serves as an ideal biomarker in other diseases. Lv et al profiled the specific urinary cell-free eccDNA (ucf-eccDNA) landscape in patients with advanced chronic kidney disease, revealing more ucf-eccDNAs in the patient urine and the enrichment of eccDNAs carrying miRNA genes (*i.e.*, MIR3200, MIR6806, MIR4508).^64^ A study explored the characteristics of plasma eccDNAs in patients with chronic schizophrenia and identified eccDNA carrying the *TAOK2* gene as a potential noninvasive biomarker for diagnosing and monitoring schizophrenia.^15^ Similarly, there are several differential eccDNA molecules in osteoporotic bone tissues compared with normal bone tissues.^93^ From the plasma eccDNA landscape in patients with type 2 diabetes mellitus, Xu et al found that eccDNAs carrying metabolism-related genes were unevenly distributed between hypoglycemic and hyperglycemic patients, and that cell-free eccDNA in plasma correlated with both glycemia changes and long-term glycemic control.^94^

Tuberous sclerosis, a multisystem genetic disorder characterized by benign lesions in various organs,^95^ shows more and longer eccDNAs in angiofibroma-derived cell cultures than in normal skin cultures.^96^^,^^97^ Besides, the amount of eccDNA increased with age in angiofibroma-derived cell cultures.

eccDNA is implicated in the pathological process of aging. Prada-Luengo et al demonstrated that eccDNA heterogeneity offers flexibility in adaptive evolution, and this heterogeneity is remarkably diminished with age.^37^ Hong et al generated comprehensive eccDNA datasets from the brains of young and elderly mice and linked the molecular phenotype of eccDNA with brain aging, facilitating the acquisition of aging biomarkers in mammalian brains.^98^ In addition, Gerovska et al reported distinct eccDNA features in skeletal muscles of sedentary versus physically active aged people.^46^

## The clearance mechanism of eccDNA

For large-scale application of eccDNA biomarkers, it is crucial to understand the clearance mechanism of eccDNA. Comparative studies in cultured cells demonstrated that eccDNA decay kinetics were nearly identical to those observed for standard plasmid and linear DNA, suggesting analogous intracellular clearance mechanisms.^99^ The average half-life of fetal eccDNA in the maternal blood is about 29.7 min 32. Sin et al established a biological link between nucleases and eccDNA properties, revealing that DNASE1L3 could digest extracellular eccDNA without affecting intracellular eccDNA.^89^ In addition, the filtration of eccDNA from plasma into the urinary system may represent another elimination route.^64^

## Conclusions

These in-depth eccDNA studies have provided novel insights into disease biomarker discovery. However, there are still some challenges in the process of moving from bench to clinical application. Before the widespread application of eccDNA biomarkers in clinical practice, the standardization of the eccDNA detection workflow is a critical issue that urgently needs to be addressed. Additionally, no method can selectively remove harmful eccDNAs. The junction position of eccDNA represents its most distinctive feature compared with chromosomal DNA, so it is feasible to design a scheme based on the junction position, while ensuring that chromosomes are not damaged. In liquid biopsy, eccDNA features in body fluids other than blood and urine also need to be explored. There is an urgent need for more evidence and clinical data to support the clinical relevance of eccDNA, which may require multi-center retrospective and prospective studies.

In summary, these discoveries of eccDNA at the basic and clinical levels have brought us new insights into disease biomarkers and may lead to robust advancements in disease diagnosis and treatment.

## CRediT authorship contribution statement

**Xuanmei Luo:** Writing – original draft, Validation, Software. **Jian Cui:** Methodology, Investigation. **Hexin Li:** Software. **Gang Zhao:** Writing – review & editing, Supervision, Funding acquisition. **Lihui Zou:** Writing – review & editing, Supervision, Funding acquisition.

## Funding

This work was supported by the National High Level Hospital Clinical Research Funding (China) (No. BJ-2024-095, BJ-2023-077).

## Conflict of interests

The authors declared no conflict of interests.
